# Body temperature and esthesia in individuals with stroke

**DOI:** 10.1038/s41598-021-89543-3

**Published:** 2021-05-12

**Authors:** Caren da Silva Dias, Fábio Marcon Alfieri, Artur Cesar Aquino dos Santos, Linamara Rizzo Battistella

**Affiliations:** 1 Institute of Physical Medicine and Rehabilitation, University of Sao Paulo School of Medicine, São Paulo, São Paulo Brazil; 2 Clinical Research Center, Institute of Physical Medicine and Rehabilitation, University of Sao Paulo School of Medicine, Rua Domingo de Soto 100, Vila Mariana, São Paulo, SP 04116-030 Brazil; 3 Health Promotion, Adventist University of São Paulo (UNASP), São Paulo, Brazil; 4 Professora Titular da Faculdade de Medicina FMUSP, University of São Paulo, São Paulo, Brazil

**Keywords:** Neuroscience, Physiology, Diseases, Neurology, Materials science

## Abstract

Patients with sequelae of stroke commonly report somatosensory losses. It is believed that body temperature may be associated with tactile sensibility and sensorimotor recovery of these patients. Demonstrate the associations among tactile sensibility, cutaneous temperature, subjective temperature perception, and sensorimotor recovery of patients with stroke sequelae. 86 patients with stroke sequelae were included. Patients had standardized regions of interest (ROIs) assessed with infrared thermography (FLIR T650SC) and monofilaments esthesiometry, and global motor recovery was evaluated with Fugl-Meyer Assessment (FMA). The presence of self-reported perception of temperature difference was used to divide the participants into two groups of 43 patients, and correlation tests were applied to establish correlations among variables. There is no clinically relevant association between tactile sensibility and cutaneous temperature of the foot, regardless of the subjective sensation of temperature changes. Sensorimotor recovery evaluated by FMA is associated with the difference of sensibility between both sides of the body (p < 0.001), as well as with the difference of tactile sensibility (p < 0.001). A clinically significant association between the difference of cutaneous temperature and tactile sensibility was not found, regardless of the presence or absence of subjective perception of such temperature difference. However, sensorimotor recovery is correlated with cutaneous temperature differences and tactile sensibility.

## Introduction

Patients with sequelae of cerebral vascular accident (CVA or Stroke) may experience several symptoms, among which the sensory deficit, a sequela that may affect more than 50% of patients with stroke^1,2^. The loss of perception, interpretation, or integration of sensory information may significantly affect the manual skills, safety, and ability, hindering daily living activities and causing the negligence of the affected limb in the functional performance and sensorimotor recovery. Among the sensorimotor alterations, the sensation is the patient's interpretation of the stimuli their senses have captured. In contrast, sensibility is the patient perception towards an external stimulus, such as tactile sensibility, which will be investigated in this study^1,2^.

A detailed investigation of somatosensory changes is critical to provide the best physical rehabilitation strategy^1–3^. As the plantar afference region contains the only receptors that touch the ground during gait or orthostatism, this region requires double the concern in an evaluation process^4^. Plantar sensibility may be assessed by esthesiometry of nylon monofilaments^5–7^.

Patients with stroke commonly report the sensation of colder temperature in the plegic limb^8^. A study reported that 64% of patients with stroke sequelae reported this alteration in thermic sensation. Indeed, the plegic upper limb may be 1–5 °C colder when compared to the contralateral limb^9^.

The temperature asymmetry between the plegic and the contralateral sides of limbs may be measured by infrared thermography^10,11^. This technique allows the recording of energy irradiated by the patient's body and provides information on physiologic responses by reading the skin temperature^12^, whose distribution between both hemispheres should be symmetric^10,13–16^.

Although this thermic sensation is reported as unpleasant by patients with stroke^17^, this issue is still scarcely explored. Some researchers state that temperature alteration after stroke may be explained by spinothalamic implications, disturbances in the medial lemniscus, and injuries in central autonomic pathways. Such issues may cause dysfunctions in vasomotor regulation^8^.

Infrared thermography of cutaneous temperature of patients with stroke showed a real asymmetry, especially in their feet, where the plegic side was colder than the contralateral limb. Also, it was observed that age, time since stroke onset, body mass index, and motricity did not influence this temperature alteration^8^.

We believe that body temperature may be associated with tactile sensibility and sensorimotor recovery of patients with stroke, which may substantially contribute to understanding this subject. Therefore, our objectives were to establish the association of tactile sensibility and body temperature of standardized regions of interest (ROIs). We also proposed identifying temperature and tactile sensibility differences demonstrating their association with sensorimotor recovery and studying the influence of the subject's perception of body temperature differences in association with tactile sensibility.

## Methods

This is a cross-sectional study, conducted at the *Instituto de Medicina Física e Reabilitação (IMREA), do Hospital das Clínicas da Faculdade de Medicina da Universidade de São Paulo*, a rehabilitation facility. This study was approved by the Institutional Ethics Review Board (Comitê de Ética para Análise de Projetos de Pesquisa—CAPPesq), registration number 2.335.371. All of the investigations complied with national law and with the Declaration of Helsinki. Written informed consent was obtained from all individual patients included in the study.

Individuals of both sexes, aged 18 years or older, with 3–36 months after stroke episode, were included in this study. Impossibility to maintain orthostatism, presence of severe cognitive disorders that hindered the comprehension of evaluations, peripheral nervous or diseases, tumors, and systemic autoimmune diseases were the exclusion criteria.

After recruitment and consent processes, 86 patients were included and divided into two groups: one group of 43 patients sensation of temperature alteration in the plegic side and 43 patients without. The sample size was estimated considering that if the proportion of individuals with tactile sensibility alterations is about 60% among the stroke population^18^, we considered that such proportion would be 80% among those who reported the subjective sensation of temperature alteration in the plegic side. Also, the sample size calculation considered α = 0.05 and power = 0.80.

After inclusion, the demographical and clinical characteristics such as age, sex, body mass index (BMI), time after stroke, length of physical rehabilitation, blood pressure, and heart rate of the patients were assessed with a visual analog scale (VAS), the Fugl-Meyer Assessment (FMA), monofilaments esthesiometry and infra-red thermography.

The Visual Analogue Scale was applied so that the patients could describe, on a continuous scale of 1–10, how much temperature alteration they experienced in their plegic side of upper limbs (UL) and lower limbs (LL), as compared to the contralateral side^19^.

The Fugl-Meyer Assessment was used to evaluate their sensory-motor recovery on all subscales: passive mobility, pain, sensibility, motor function of upper and lower limbs, and balance^20,21^. In the FMA, each item is rated from 0 to 2, lower scores mean more significant sensory-motor disability, and the total score is 226. This score quantifies the degree of sensory-motor impairment and can be categorized as severe (50 points or lower), significant (51–84 points), moderate (85–95 points), and slight (above 96 points)^21^.

The plantar region's tactile sensibility was evaluated with the Semmes–Weinstein monofilaments esthesiometry, a gold standard quantitative test to evaluate the tactile sensory performance^4–7^. The objective of this evaluation was to test the degree of sensibility towards standardized touch and pressure on the plantar skin^7,22^.

The monofilaments were tested in an ascending fashion, from the smallest to the largest caliber, and the analysis was conducted according to their classification of colors: green and blue, normal sensibility; purple, diminished discrimination of temperature and form; red, diminished protective sensation, vulnerability to injuries; orange, loss of protective sensation; and pink, total sensibility loss, total protective sensation loss, deep pressure sensation only. A numeric score was defined to each color, so that comparative analysis could be conducted (green = 1, blue = 2, purple = 3, red = 4, orange = 5, and pink = 6). Both feet were assessed as previously described by Ueda and Carpes^7^. In each foot, nine regions were evaluated: 1—the digital surface of the fifth distal phalanx; 2—the digital surface of the third distal phalanx; 3—the digital surface of the first distal phalanx; 4—metatarsal head of the fifth phalanx; 5—metatarsal head of the third phalanx; 6—metatarsal head of the first phalanx; 7—the lateral region of the midfoot; 8—the medial region of the midfoot; and 9—calcaneus plantar region. The same evaluator conducted all evaluations in the same room under similar environmental conditions, and the patients were lying down with their eyes closed^7^.

The body temperature was measured by infrared temperature. This evaluation was conducted in the Thermography Laboratory of the *Instituto de Medicina Física e de Reabilitação (IMREA) do Hospital das Clínicas da Faculdade de Medicina da Universidade de São Paulo (HCFMUSP)*. As this evaluation must follow standardized procedures, we applied the conditions and actions described in the literature^12,16,23–28^. The laboratory had an average temperature of 21 °C, the windows, shutters, and doors were closed so that external light did not interfere in the infrared thermography. The relative humidity was 65% on average. All evaluations were conducted in the morning.

### Patient preparation

The patients were instructed not to take a bath or hot showers, use ointments or powders, or to join vigorous physical exercises two hours before evaluations. They should not eat within the two hours before the assessment and were asked not to ingest stimulating substances, caffeine or alcoholic drinks, not smoke, and not to use any nasal decongestant. At last, the patients were acclimatized for 20 min in the thermography lab so that the skin reached a thermic balance with the environment. Patients were not evaluated if there were signs of infection, such as fever^8,12,16,28^.

### Images collection and analysis

**Figure 1:**
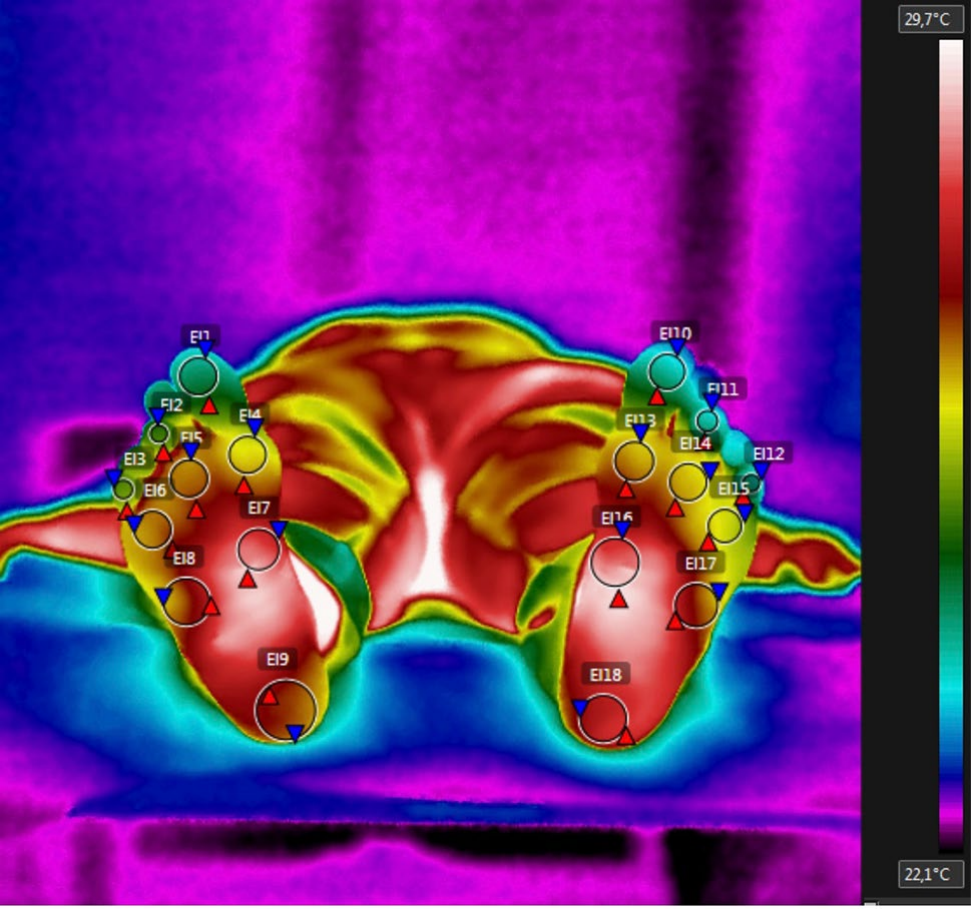
Thermographic image of the regions of interest (ROIs) in the foot. Figure obtained by the FLIR Tools software (https://www.flir.com.br/products/flir-tools/).

Initially, the patients had infrared images of both feet' plantar regions recorded. They were asked to lay on dorsal decubitus with their feet on dorsiflexion, and perpendicular images of the whole plantar surface were collected. The Regions of Interest (ROIs) were the calcaneus, lateral and medial regions of the midfoot, digital surface of the fifth, third and first toes, and first, third, and fifth metatarsal regions. As described by Gatt et al., the infrared camera was placed 1.5 m from the surfaces to be recorded^29^ (Fig. 1).

The thermographic images were captured by a FLIR T650SC infrared sensor, with a resolution of 640 × 480 pixels, at a frequency of 30 Hz. This sensor can collect images with a temperature range from – 40 to 70 °C, with a precision of 1%, and a spectral band of 7.4–14 µm, NETD < 20 mK. The thermic sensibility of 0.03 °C was used with a colorimetric scale (color palette), considering a skin emissivity of 0.98^30^. Both anterior and posterior incidences of right and left sides of upper and lower limbs were collected. All images were analyzed with FLIR Tools software.

**Figure 2:**
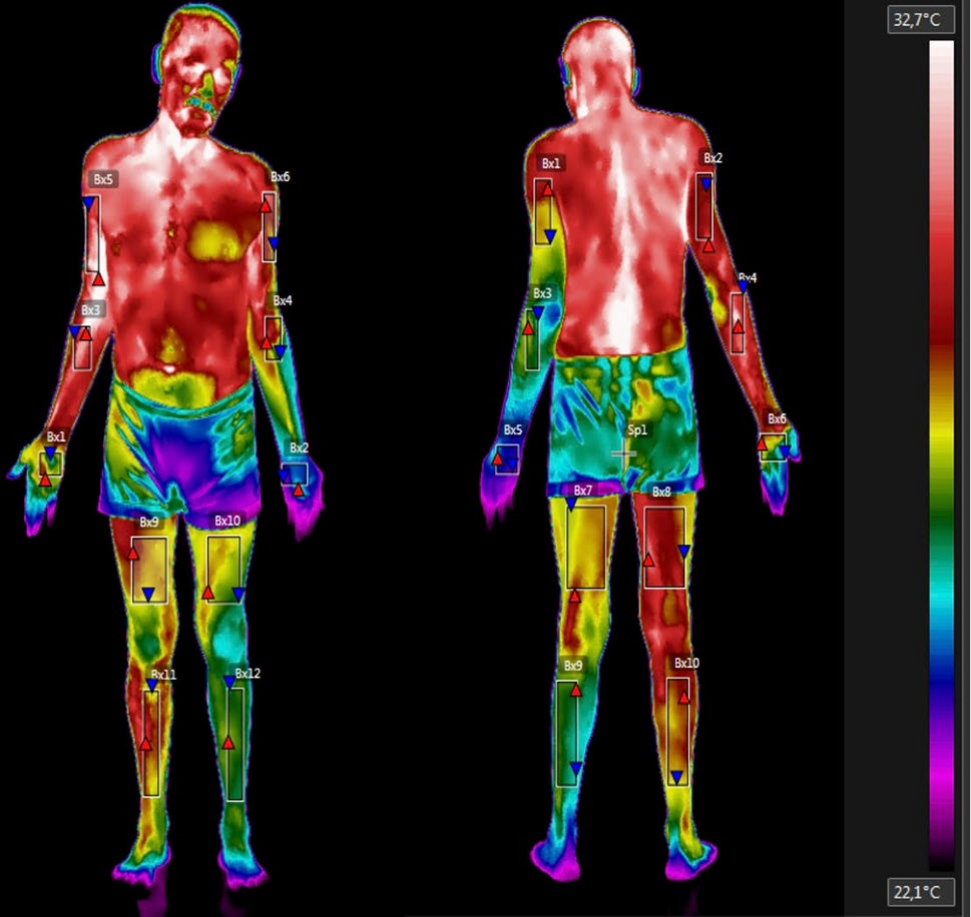
Thermographic image of the regions of interest (ROIs) of whole body. Figure obtained by the FLIR Tools software (https://www.flir.com.br/products/flir-tools/).

The temperature scale used was Celsius, and the data under analyses were the mean temperature of each ROI. Each ROI was standardized rectangles within anatomical regions, as follows: 1—hand, the junction of the third metacarpal with the third proximal phalanx and the cubital styloid process; 2—forearm, distal forearm, and cubital fossa; 3—arm, cubital fossa and axillar line; 4—thigh, 5 cm above the upper bond of the patella and the inguinal line; 5—leg, 5 cm below the lower bond of the patella and 10 cm above the malleolus. This evaluation was also described in other studies^9,12^ (Fig. 2).

The temperature was recorded in Celsius degrees, and the mean temperature of each ROI was the variable under statistical analyses were.

### Data analysis

The results were primarily presented as means ± standard deviations. The normality of the variables was tested with Shapiro–Wilk. The tactile sensibility of the body's plegic side was subtracted from the contralateral side, generating a categorical score of 0–5 points of the intensity of tactile difference between both sides of the body.

These categorical data were tested in association with temperature differences between both sides of the body with Spearman's rank correlation test. This approach was applied to establish whether the increased difference of tactile sensibility is associated with more significant temperature differences.

The influence of the self-reported perception of temperature differences over tactile sensibility was also tested with Spearman's rank correlation. The association of temperature differences or sensibility differences between both sides of the body and sensorimotor recovery was demonstrated with simple linear regression where sensorimotor recovery was the dependent variable.

Finally, demographic data was investigated with linear regression analysis so that their influence over the temperature and tactile sensibility variables of this study could be explored.

In all cases, the statistical significance was considered when α ≤ 0.05. The statistical analysis was conducted with Stata14.

## Results

During recruitment and screening, 128 patients were invited to participate in the study. Twenty-eight patients were excluded due to diabetes; one patient for not holding an orthostatic position, and 28 patients did not consent to participate. Then, 86 patients were included and had their data collected and analyzed.

The Shapiro–Wilk normality test evidenced temperature was not parametric. However, visual analysis of distribution and analysis kurtosis and skewness analysis evidenced otherwise. Even though the sample size was large, we decided to analyze the primary objective with non-parametrical tests.

Table 1 presents the general characteristics of the included patients with information about sex, age, stroke type, plegic side, body mass index (BMI), time since stroke event, rehabilitation length, and sensorimotor recovery.

**Table 1:** General characteristics of post-stroke patients.

	Total sample (n = 86)	With sensation (n = 43)	Without sensation (n = 43)
Sex (Fem/male)	41/45	20/23	21/22
Age (years)	53.60 ± 15.89^a^	58.97 ± 13.59^a^	48.23 ± 16.34^a^
Stroke type: I/H/B (n)	53/29/4	28/11/4	26/17/0
Plegic side: R/L (n)	43/43	21/22	22/21
BMI (kg/cm^2^)	25.26 ± 4.27^a^	25.13 ± 4.07^a^	25.39 ± 4.54^a^
Time after stroke onset (months)	19.67 ± 8.21^a^	21.30 ± 7.60^a^	18.04 ± 8.56^a^
Rehabilitation time (months)	13.06 ± 9.21^a^	14.48 ± 9.12^a^	11.65 ± 9.19^a^
Sensory-Motor recovery (FMA)	137.68 ± 50.97^a^	120.60 ± 40.11^a^	154.76 ± 55.24^a^

*I* ischemic, *H* hemorrhagic, *B* both, *R* right, *L* left, *BMI* body mass index, *kg* kilogram, *cm* centimeters, *FMA* Fugl-Meyer assessment.

^a^Mean ± standard deviation.

The patients were separated into two groups, with and without sensation of temperature alterations, of 43 patients. The averages of VAS that assessed how intense was the self-reported sensation of temperature alteration was 4.9 ± 2.6 and 4.2 ± 2.7 for the upper limbs and the lower limbs, respectively.

The mean age of those with the sensation of temperature alterations was above those without this sensation. In contrast, sex, plegic side, BMI, time after stroke onset, and rehabilitation time were statistically similar between both groups. Sensory-motor recovery measured by FMA was better in the group of patients without the sensation of temperature alteration, as shown in Table 1.

Demographic variables showed no evidence of correlation with the sensation of temperature alteration or tactile sensibility. Therefore, none of these variables could be considered confounders. Age and BMI seem to be associated with temperature differences, whereas age alone is associated with tactile sensibility differences (p = 0.011, β < − 0.01; p = 0.04, β = − 0.02; and p < 0.001, β = 0.01 respectively). Nonetheless, the minuscule β coefficients showed the influence of these demographic variables should not be considered.

The temperature differences between the plegic and contralateral ROIs are not associated with tactile sensibility differences between the same regions. The Spearman's rank correlation test failed to detect such association as rho = − 0.008 and p = 0.809. Regarding the influence of the subjective perception of the temperature difference between the sides of the body, the statistical analyses have shown otherwise. When the presence of subjective perception of temperature differences is considered, there is a statistically significant but weak association between temperature and tactile sensibility, as the difference of tactile sensibility between the sides of the body slightly increases along with the increase in temperature difference. Oppositely, among those who do not report temperature alterations, the association is inverted and the difference of tactile sensibility between the plegic and the contralateral sides decreases as the temperature difference increases. Table 2 describes these results.

**Table 2:** Spearman’s correlation of temperature and tactile sensibility differences in the feet.

Association	Spearman’s rho	p-value
Temperature difference × tactile sensibility difference	− 0.008	0.809
	Group 1	
Temperature difference × tactile sensibility difference	− 0.102	0.045
	Group 2	
Temperature difference × tactile sensibility difference	0.161	0.002

Group 1: Patients without the subjective perception of temperature differences between both sides of the body; Group 2: Patients with the subjective perception of temperature differences between both sides of the body.

**Figure 3:**
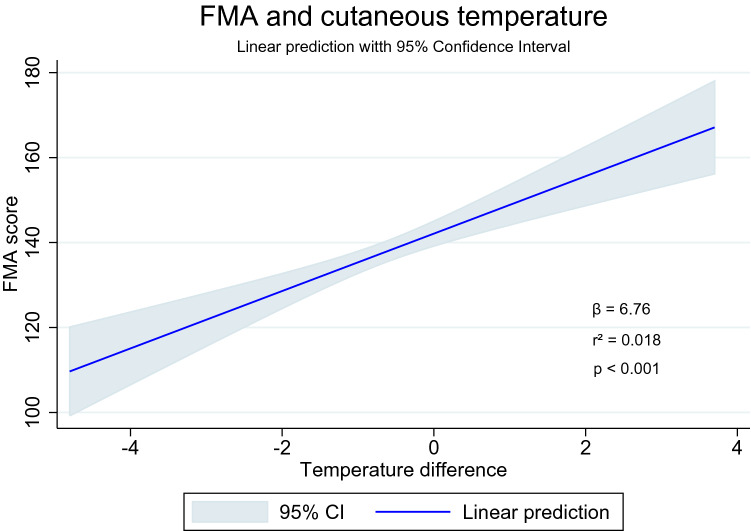
Linear prediction of sensorimotor recovery (FMA) over cutaneous temperature difference. Image obtained by the Stata14 program (https://www.stata.com/stata14/).

**Figure 4:**
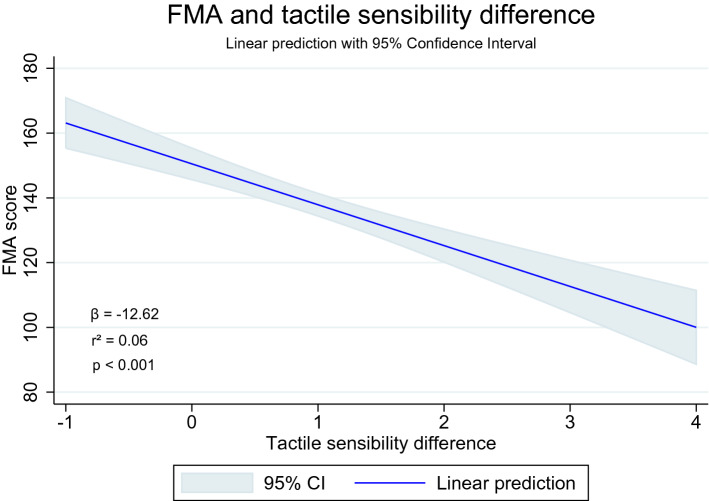
Linear prediction of sensorimotor recovery (FMA) over tactile sensibility difference. Image obtained by the Stata14 program (https://www.stata.com/stata14/).

Sensorimotor recovery assessed by FMA was associated with both temperature and tactile sensibility differences, regardless of the presence or absence of subjective perception of temperature (Figs. 3, 4). Post hoc analysis evidenced that inner domains of FMA domains of mobility were associated with temperature and tactile sensibility differences, as presented in Table 3.

**Table 3:** Association of sensorimotor recovery and temperature or tactile sensibility differences.

Regression variables	β	95% CI		p-value	r^2^
		Lower bound	Upper bound		
Temp. difference × FMA total score	6.76	4.31	9.20	< 0.001	0.018
FMA mobility	0.62	0.26	0.99	0.001	0.007
FMA pain	0.62	0.36	0.87	< 0.001	0.014
FMA exteroception	0.01	− 0.10	0.12	0.807	< 0.001
FMA proprioception	− 0.12	− 0.36	0.12	0.339	< 0.001
FMA upper limb recovery	5.09	3.84	6.35	< 0.001	0.04
FMA lower limb recover	0.93	0.44	1.42	< 0.001	0.008
FMA balance	0.04	− 0.09	0.17	0.541	< 0.001
Tactile sensibility difference × FMA total score	− 12.62	− 16.18	− 9.01	< 0.001	0.06
FMA mobility	− 1.54	− 2.07	− 1.00	< 0.001	0.04
FMA pain	− 0.58	− 0.96	0.19	0.003	0.01
FMA exteroception	− 0.85	− 1.01	− 0.70	< 0.001	0.13
FMA proprioception	− 1.67	− 2.01	− 1.33	< 0.001	0.11
FMA upper limb recovery	− 5.17	− 7.05	− 3.30	< 0.001	0.04
FMA lower limb recover	− 3.35	− 4.04	− 2.66	< 0.001	0.11
FMA balance	− 0.44	− 0.62	− 0.25	< 0.001	0.03

*β* beta coefficient of linear regression, *CI* confidence interval of beta coefficient, *Temp*. temperature, *FMA* Fugl-Meyer Assessment. Note 1: Fugl-Meyer scores were considered dependent variables; Note 2: statistical significance was achieved whenever p ≤ 0.003, due to correction for multiple analysis (p/n_analysis_, with analysis = 16); Note 3: The difference of temperature considered lower and upper limbs; Note 4: the tactile sensibility of the feet was analyzed as an independent variable.

## Discussion

The objective of this study was to demonstrate the associations among tactile sensibility, cutaneous temperature, subjective temperature perception, and sensorimotor recovery of patients stroke sequelae. These issues were addressed with the assessments of Fugl-Meyer for sensory-motor recovery, infrared imaging for quantifying the temperature of each limb, and the monofilaments esthesiometry for the cutaneous sensibility of lower limbs.

Regarding sex, the sample was homogeneous, once 52% of the patients were male and 48% female. As for age, patients were prevalently about 50 years of age, i.e., predominantly adult-elderly; therefore, this population is naturally subject to an accuracy reduction of function sensorimotor due to the aging process^3,31,32^. Comparing groups of patients with and without the sensation of temperature alterations in the plegic side evidenced a 10-year difference between them. Those with this sensation were older than those without the sensation.

Previous studies show that among stroke patients, the ischemic type is the most prevalent^33–36^, a figure that was also present in our study, once the number of patients with the diagnosis of ischemic stroke was significantly higher, reaching approximately 62% of the population studied.

Regarding BMI, Alfieri et al. studied a sample of patients with IMC around 25.4 kg/cm^2^, similar to our sample, with IMC of 25.26 kg/cm^2^ in patients with sequelae stroke^8^. Previous studies show that the ischemic type is the most prevalent among stroke patients^33–36^, which agrees with our study. The number of patients diagnosed with ischemic stroke was approximately 62% of the population studied. None of the demographic characteristics listed above was considered confounders in our study; hence we did not conduct any statistical adjustments.

In our study, the sensation of alteration in temperature was considered once it is frequently reported as an uncomfortable symptom and a relevant factor studied by previous studies^17,18,37,38^. This sensation of temperature alteration may be associated with the sympathetic fiber's autonomic dysfunction, which may interfere with the blood flow^39^. When such subjective perception is added to the analysis, there is a statistically significant association between tactile sensibility and cutaneous temperature. However, this association is very weak and can be ignored once there is no clinical significance in our findings.

We also emphasize that tactile sensibility analysis considered the cutaneous region of the feet, the only one assessed by esthesiometry and infra-red thermography concomitantly. Such ROIs are of great sensory importance, and the most significant temperature differences are found between the plegic and contralateral sides in these ROIs^4,8^. The findings of the present study agree with another study published by our group in which we found that the subjective perception of temperature changes does not correlate with the real difference of temperature found by IR thermography^8^.

Although no association between the studied variables of tactile sensibility and temperature differences was found, our results suggest that mechanoreceptors response may not be correlated with temperature control, which in turn may be correlated with the hypothalamus, which balances heat generation with heat loss, being connected to the pituitary gland at the base of the brain near to the termination of the brain stem^6,40^ or even with the degree of spasticity and strength of the patient^41^.

This previous study^8^ found that, by evaluating the thermography of both sides of the body, BMI, age, time after stroke, and motricity has no association with the difference of temperature between the plegic and the contralateral sides of patients with stroke sequelae. Even though other studies evaluated motricity with the National Institute of Health Stroke Scale (NIHSS)^21,42^, we decided to apply the FMA to assess our patient's recovery, given its important correlation with sensibility.

Oppositely to Alfieri et al.^8^, we found a significant association between the motor-recovery and tactile sensibility temperature. Our findings show that a more significant sensorimotor recovery is found the closer the difference in temperature or tactile sensibility is to zero, regardless of the patient's subjective perception of temperature. We consider this association essential once it establishes that temperature and tactile sensibility are relevant issues to be considered for the rehabilitation of patients with stroke.

Our results are relevant because our findings help solve rehabilitation professionals and patients' concern that a more significant sensorimotor recovery means smaller temperature differences. Our results assure the relevance of how important body temperature is as a measure of the outcome of the treatment of patients with stroke sequelae. An example is a study that assessed the effect of massage and foot baths on psychophysiological responses of patients with stroke. This study showed that patients assigned to the experimental group had greater satisfaction towards their treatment and better temperature balance between both sides of the body^43^. Zanona et al. investigated this subject when they combined Occupational Therapy and Virtual Reality for treating functionality and body temperature of patients with stroke sequelae^44^. Other examples are found, such as the publication that reported increased temperature in the affected limb after a rehabilitation program, allowing the researchers to establish a strong correlation between improvement of joint function and temperature changes^45^.

Our sample was composed of patients who received physical rehabilitation treatment at the same facility, with similar programs and rehabilitation length. Also, we emphasize that the evaluations were all conducted by the same evaluator. However, no information on the dominant side, presence of spasticity, and size and location of brain injury were available, variables that must certainly be investigated in future studies. Nonetheless, this study's objective focused on analyzing the correlation between cutaneous sensibility and sensorimotor recovery. Therefore, we understand that, despite being limitations, they did not interfere with our results.

Even though, to the best of our knowledge, there is no standard rehabilitation protocol in the literature to improve the difference in temperature perception post-stroke, we believe this issue should be taken into consideration since these patients commonly report this complaint. Still, future studies assess the recovery of individuals associated with the distribution of body temperature and consider factors such as the use of medications that may affect, for instance, spasticity.

## Conclusions

The results of this study allow the conclusion that even though there is a statistically significant correlation between tactile sensibility and cutaneous temperature differences in patients with stroke, this association is neglectable and clinically irrelevant. Nonetheless, there is a significant association between sensorimotor recovery and differences in cutaneous temperature and differences in tactile sensibility between both sides of the body.
